# The Virtual Brain: a neuroinformatics platform for simulating large-scale brain network models

**DOI:** 10.1186/1471-2202-14-S1-P193

**Published:** 2013-07-08

**Authors:** Paula Sanz Leon, Marmaduke Woodman, Randy McIntosh, Viktor Jirsa

**Affiliations:** 1 Institut de Neuroscience des Systemes, UMR1106, Aix Marseille Universite , Marseille, France; 2 Rotman Research Institute of Baycrest Centre, University of toronto, Ontario, Canada

## 

*TheVirtualBrain *(TVB) is the first integrative neuroinformatics platform for the modeling of full brain network dynamics. TVB simulator, written in Python, enables the systematic model-based inference of neurophysiological mechanisms on different brain scales that underlie the generation of macroscopic commonly used neuroimaging signals (EEG, MEG, fMRI). In the framework of TVB we build full brain network models by incorporating biologically realistic large scale couplings of neural populations. The couplings within the network are captured by the space-time structure of the connectivity matrix, which defines the connection strengths and time delays via signal transmission between all network nodes. Researchers from computational, theoretical and clinical neuroscience can benefit of this tool. The web interface allows users without programming knowledge to access a powerful computing platform as well as visualization and analysis tools. Users with strong programming skills benefit of all the advantages provided by the Python programming through a shell interface.

## Conclusions

We propose TVB as a novel computing tool for large-scale brain simulations. TVB is the solution to tackle systematic parameter space exploration, allowing to rapidly gain insights of the full brain dynamics repertoire as a function of structure. It moves away from the investigation of isolated regional responses and considers the function of each region in terms of the interplay among brain regions. Full brain simulations open the possibility for the exploration of the consequences of pathological changes in the system, enabling us to identify and potentially counteract those unfavorable processes.

**Figure 1:**
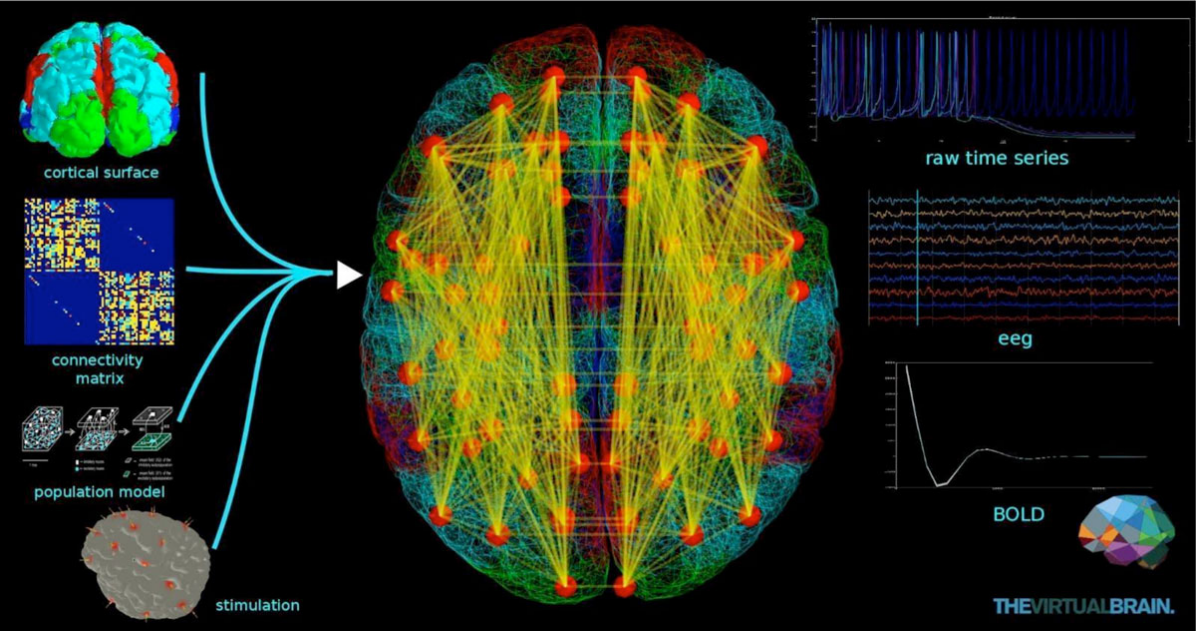
**Schematic representation of a brain network model in TVB**. Together, the cortical surface and its local connectivity, the connectivity matrix and the neural mass models at the network nodes define The Virtual Brain. Additionally, stimulation patterns can be applied during simulations. Output time-series correspond to commonly used neuroimaging recordings such as EEG, MEG and BOLD.

